# SlZF3 regulates tomato plant height by directly repressing *SlGA20ox4* in the gibberellic acid biosynthesis pathway

**DOI:** 10.1093/hr/uhad025

**Published:** 2023-02-21

**Authors:** Jinying Luo, Yunfei Tang, Zhuannan Chu, Yuxin Peng, Jiawei Chen, Huiyang Yu, Chunmei Shi, Jahanzeb Jafar, Rong Chen, Yaping Tang, Yongen Lu, Zhibiao Ye, Ying Li, Bo Ouyang

**Affiliations:** National Key Laboratory for Germplasm Innovation & Utilization of Horticultural Crops, College of Horticulture and Forestry Sciences, Huazhong Agricultural University, Wuhan 430070, China; National Key Laboratory for Germplasm Innovation & Utilization of Horticultural Crops, College of Horticulture and Forestry Sciences, Huazhong Agricultural University, Wuhan 430070, China; National Key Laboratory for Germplasm Innovation & Utilization of Horticultural Crops, College of Horticulture and Forestry Sciences, Huazhong Agricultural University, Wuhan 430070, China; National Key Laboratory for Germplasm Innovation & Utilization of Horticultural Crops, College of Horticulture and Forestry Sciences, Huazhong Agricultural University, Wuhan 430070, China; National Key Laboratory for Germplasm Innovation & Utilization of Horticultural Crops, College of Horticulture and Forestry Sciences, Huazhong Agricultural University, Wuhan 430070, China; National Key Laboratory for Germplasm Innovation & Utilization of Horticultural Crops, College of Horticulture and Forestry Sciences, Huazhong Agricultural University, Wuhan 430070, China; National Key Laboratory for Germplasm Innovation & Utilization of Horticultural Crops, College of Horticulture and Forestry Sciences, Huazhong Agricultural University, Wuhan 430070, China; National Key Laboratory for Germplasm Innovation & Utilization of Horticultural Crops, College of Horticulture and Forestry Sciences, Huazhong Agricultural University, Wuhan 430070, China; National Key Laboratory for Germplasm Innovation & Utilization of Horticultural Crops, College of Horticulture and Forestry Sciences, Huazhong Agricultural University, Wuhan 430070, China; National Key Laboratory for Germplasm Innovation & Utilization of Horticultural Crops, College of Horticulture and Forestry Sciences, Huazhong Agricultural University, Wuhan 430070, China; National Key Laboratory for Germplasm Innovation & Utilization of Horticultural Crops, College of Horticulture and Forestry Sciences, Huazhong Agricultural University, Wuhan 430070, China; National Key Laboratory for Germplasm Innovation & Utilization of Horticultural Crops, College of Horticulture and Forestry Sciences, Huazhong Agricultural University, Wuhan 430070, China; College of Horticulture, Henan Agricultural University, Zhengzhou 450002, China; National Key Laboratory for Germplasm Innovation & Utilization of Horticultural Crops, College of Horticulture and Forestry Sciences, Huazhong Agricultural University, Wuhan 430070, China

## Abstract

Plant height is an important target trait for crop genetic improvement. Our previous work has identified a salt-tolerant C2H2 zinc finger, SlZF3, and its overexpression lines also showed a semi-dwarf phenotype, but the molecular mechanism remains to be elucidated. Here, we characterized the dwarf phenotype in detail. The dwarfism is caused by a decrease in stem internode cell elongation and deficiency of bioactive gibberellic acids (GAs), and can be rescued by exogenous GA3 treatment. Gene expression assays detected reduced expression of genes in the GA biosynthesis pathway of the overexpression lines, including *SlGA20ox4*. Several protein–DNA interaction methods confirmed that SlZF3 can directly bind to the *SlGA20ox4* promoter and inhibit its expression, and the interaction can also occur for *SlKS* and *SlKO*. Overexpression of *SlGA20ox4* in the *SlZF3*-overexpressing line can recover the dwarf phenotype. Therefore, SlZF3 regulates plant height by directly repressing genes in the tomato GA biosynthesis pathway.

## Introduction

Plant height is one of the most important agronomic traits in crops, which is especially evident in the Green Revolution in cereal crops. It affects crop architecture, land utilization, nutrition, and production management [1]. Plant height is controlled by many genes, especially those related to plant hormone biosynthesis or signal transduction, including gibberellic acid (GA), auxin, brassinosteroid, and strigolactone [2–5]. The Green Revolution in the 1960s was largely a result of the successful introduction of high-yield and semi-dwarf wheat and rice varieties [6–8]. Molecular genetic studies have shown that Green Revolution genes are closely related to gibberellins, such as rice *SD1*, encoding GA20 oxidase in rice GA biosynthesis, and wheat *Rht1*, encoding DELLA protein in GA signal transduction [6, 7].

GA biosynthesis has been extensively studied in plants. GAs belong to the tetracyclic diterpenoid acids, which affect the growth and development of plants throughout their life cycle [9]. More than 130 GA compounds have been identified, among which GA1, GA3, GA4, and GA7 are bioactive forms [10]. The biosynthesis of GAs is a three-step process [11, 12]. First, *ent*-copalyl diphosphate synthase (CPS) and *ent*-kaurene synthase (KS) participate in the synthesis of *ent*-kaurene from geranylgeranyl diphosphate (GGPP) [13, 14]. Second, three enzymes, including cytochrome P450 enzymes, *ent*-kaurene oxidase (KO), and *ent*-kaurenoic acid oxidase (KAO), are involved in the conversion of *ent-*kaurene into GA12 [15, 16]. Third, all common GA precursors are converted to bioactive GAs by GA20ox and GA3ox (GA3 oxidase). Gene defects involved in the first/second step of GA biosynthesis usually lead to severe dwarfism and impaired growth, and can be rescued by exogenous GA3 [17–20]. However, mutants of the last-step genes often display a semi-dwarf phenotype because of gene functional redundancy, such as GA3oxs in *Arabidopsis*, maize, and rice [7, 21–23]. Typically, the rice Green Revolution gene *SD1* encodes a GA20 oxidase [7]. In *Arabidopsis*, *GA20ox1* and *GA20ox2* function partially redundantly, and the mutation results in a semi-dwarf phenotype [23]. Loss of function of *OsGA3ox2* also results in the semi-dwarf phenotype of rice *d18* mutant [21].

Many transcription factors have been revealed to participate in regulating plant height via the GA biosynthesis pathway. In *Arabidopsis*, the NAC-like protein GSF upregulates *AtGA2ox2* to reduce the GA level, and overexpression of *GSF* causes dwarfism that can be rescued by external GA3 application [24]. The transcription factor MYB62 can directly bind to the promoter of *AtGA2ox7* and upregulate its expression to affect plant height [25]. Overexpression of *AtWOX14* results in the accumulation of bioactive GAs by activating GA3oxs and suppressing GA2oxs [26]. In rice, OsWOX3A directly represses the transcription of *KAO*, leading to severe dwarfism [27]. Rice OsNAC2 was found to inhibit the expression of *KO2* and negatively regulate plant height [28]. Besides, OsMADS57 can directly inhibit the expression of *OsGA2ox3* and *EUI1* by binding the CArG-box element in the promoter, thus regulating rice plant height [29]. Most recently, the chromatin remodeling factors OsSWC4 and OsYAF9 were found to be involved in the deposition of H2A.Z and acetylation of H4 for GA biosynthesis genes by binding to the AT-rich region of the promoters, thus mediating their transcription level to promote rice internode elongation [30]. Although there are many transcription factors identified to regulate plant height, only a few were identified in the regulation of plant height in tomato or other Solanaceae crops. In tobacco, the bZIP transcription factor RSG can bind to the promoter of *NtKO* and *NtGA20ox1* via RSG-binding element, involving cell elongation and plant height. However, RSG regulates the transcription of *NtGA20ox1* through feedback regulation depending on the endogenous GA level, but RSG activates *NtKO* independently of GA concentration [31–33]. Our work in tomato shows that SlDREB can inhibit the elongation of internodes by suppressing the expression of GA genes. SlDREB can bind to the typical DRE/CRT elements in the promoter of *SlCPS* [34]. Besides, knockdown of a JmjC domain-containing protein, JMJ524, causes a GA-insensitive dwarfism in tomato. JMJ524 participates in GA signal transduction by regulating the expression of *SlGLD1* (DELLA-like gene) via DNA methylation [35]. Nevertheless, the molecular mechanism underlying tomato plant height remains largely unclear. It is expected that many other transcription factors, such as zinc fingers, are involved in the regulation of tomato plant height.

Zinc-finger proteins play important roles in plant development and stress tolerance, and are one of the largest transcription factor families in plants [36–39]. Some of them were reported to regulate the development of leaves, root hairs, and petals in plants [40–42]. However, few of them have been documented to regulate the height of plants. The C2C2 zinc fingers OsYABBY1 and OsYABBY4 can affect plant height by negatively regulating the expression of *OsGA3ox2* and *OsGA20ox2*, respectively [43, 44]. The rice C2H2 protein ZFP207 in rice acts as a repressor of *SD1* (*OsGA20ox2*) by promoter binding and affects plant height and grain length [45]. A20/AN1-type zinc finger OsSAP8 can bind to the *OsKO2* promoter via ACGTGTC element to regulate rice height. Exogenous GA3 can rescue the semi-dwarf phenotype of *OsSAP8* overexpression lines [46]. Fan *et al*. [47] found that overexpression of *SlRBZ* (RanBP2-type zinc finger gene) causes severe dwarfism by impairing GA biosynthesis in tomato, but the exact mechanism is still not clear. As far as we know, the mechanism by which zinc-finger transcription factors regulate tomato plant height is rather poorly understood, although some transgenic plants show different degrees of dwarfing [42, 48, 49].

In our previous study, we found that a nuclear zinc finger protein, SlZF3, interacts with SlCSN5B to promote the biosynthesis of ascorbic acid, thus enhancing the salt tolerance of tomato plants by reactive oxygen species (ROS) elimination [50]. Besides being involved in stress tolerance, we also found that overexpression of *SlZF3* resulted in dwarfism in tomato. Here, we found that SlZF3 regulates tomato plant height by directly binding to the promoter of GA genes, such as *SlGA20ox4*. Our work on SlZF3 suggests that it plays a coordinating role in plant defense and growth.

## Results

### Tissue and induction expression profile of *SlZF3*

Previously, *SlZF3* was found to regulate tomato salt tolerance via interacting with the COP9 subunit CSN5B and promoting the VTC1/GMPase, thus increasing the ROS scavenging capability [50]. Interestingly, overexpression of *SlZF3* also causes dwarfism of tomato plants. However, the molecular mechanism underlying this phenotype remains to be elucidated. We firstly investigated the expression characteristics of *SlZF3* using RT–qPCR.

**Figure 1:**
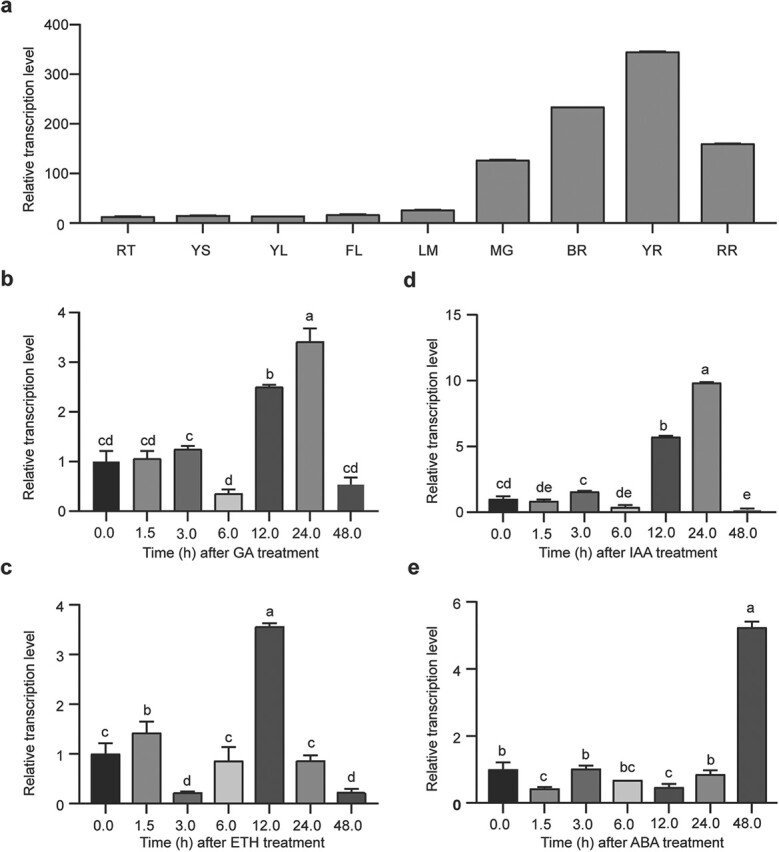
Transcription level of *SlZF3* in various tissues and response to hormone treatments in tomato. **a** Expression profile of *SlZF3* in various tissues of ‘Ailsa Craig’ (AC) tomato. RT, root; YS, young stem; YL, young leaf; FL, flower; IM, immature fruit; MG, mature green fruit; BR, breaker fruit; YR, yellow red fruit; RR, red ripe fruit. **b**–**e** Expression of *SlZF3* under treatment with 100 μM gibberellin (GA3) (**b**), 1% (v/v) ethephon (ETH) (**c**), 100 μM IAA (**d**), and 100 μM ABA (**e**). Six-week-old AC seedlings were sprayed with the hormone solutions, with water as the control. The horizontal axis shows hours after treatments. Zero (0) hour represents the untreated control. Values are mean ± standard deviation (*n* = 3). Data were submitted to one-way ANOVA and Duncan’s multiple range test. Columns followed by the same letter are not significantly different at *P* < .05.

Based on the results, *SlZF3* was constitutively expressed in the tissues we tested, and its expression was relatively high in fruits at different developmental stages (Fig. 1a). Many studies have shown that dwarfing is frequently related to plant hormones, such as GA [6, 7, 19]. Therefore, we investigated the expression of *SlZF3* under treatment with different hormones, including GA3, indoleacetic acid (IAA), ethephon (ETH), and abscisic acid (ABA)*.* It was found that *SlZF3* responded to the four hormones tested. Under GA3 and IAA, *SlZF3* showed a similar expression pattern, being significantly upregulated at 12 and 24 h but downregulated at 6 and 48 h (Fig. 1b and d). Under ETH treatment, the transcripts were significantly upregulated at 12 h but downregulated at 3 and 48 h (Fig. 1c). Under ABA treatment, *SlZF3* was repressed at most time points tested; however, it showed ~5-fold upregulation at 48 h (Fig. 1e). In short, *SlZF3* is constitutively expressed in different tissues, and the expression in leaves can be induced by different hormones, including GA.

### Semi-dwarf phenotype of *SlZF3*-overexpressing tomato lines

**Figure 2:**
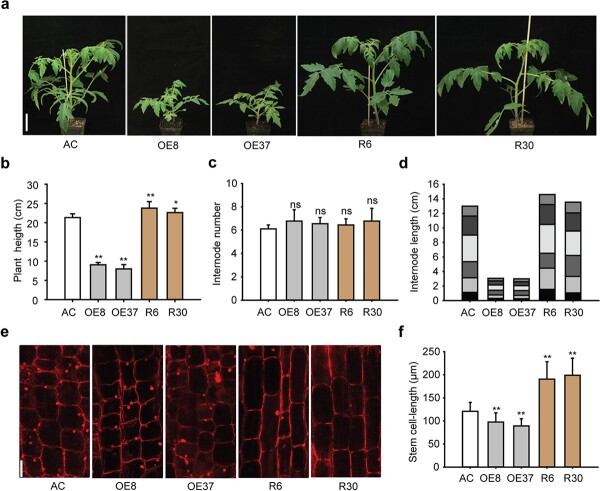
Overexpression of *SlZF3* restricts internode elongation of tomato plants. **a** Seedling phenotype of overexpression lines (OE8, OE37), RNAi lines (R6, R30), and wild-type (AC). Photographs were taken of 1-month-old seedlings. Scale bar = 5 cm. **b**–**f** Plant height (**b**), number of internodes (**c**), length of internodes (**d**), PI-stained cells in the third internode from the top of plants (**e**), and length of stained cells (**f**). Scale bar in **e** = 100 μm. Values are mean ± standard deviation, Student’s *t*-test (*n* = 9). ^*^P < .05; ^**^P < .01; ns, no significant difference.

An initial observation revealed that the *SlZF3*-overexpressing lines are semi-dwarf. We checked both the overexpression and RNAi lines of *SlZF3* in 6-week-old plants. The overexpression lines were significantly shorter than the wild-type ‘Ailsa Craig’ (AC), whereas the RNAi lines were taller than wide type (Fig. 2a). For 6-week-old seedlings, the wild-type was 21.3 cm in height, whereas the height of the overexpression lines was only ~40% of that of the wild-type. Meanwhile, the RNAi lines were clearly higher (Fig. 2b). The change in plant height could be due to the change in internode number or internode length [27, 29]. We further checked these two parameters for all the lines tested. No difference was found in the number of internodes (Fig. 2c); however, the internode length of the overexpression lines was much shorter than that of the wild-type (Fig. 2d). To further check whether the short internode resulted from a decrease in cell length, we selected the fourth internode from the bottom and examined the middle part by microscope. The results showed that, compared with AC, the length of stem cells in overexpressing lines was significantly reduced, but increased in the RNAi lines (Fig. 2e and f). Therefore, the alteration in stem cell length is a main reason for the plant height change in *SlZF3* transgenic lines.

In addition, obvious changes could also be observed in the leaves of the transgenic lines (Supplementary Data Fig. **S1**a). We found that the leaf length and width of the overexpression lines were obviously smaller than those of AC (Supplementary Data Fig. **S1**b and c). It was reported that leaf development was strongly correlated with GA levels. Leaf length can be regulated by the OsGRF7 (growth regulating factor 7)- and OsGRF8-mediated GA pathway [51], and exogenous GA3 can promote greater leaf area and thinner leaves [52]. Paraffin sections showed that the leaves of overexpressing plants were significantly thicker than those of AC, but were thinner for the RNAi lines (Supplementary Data Fig. **S1**d and e). These results indicated that *SlZF3* is a pleiotropic gene that controls diverse biological processes.

### Reduced level of GA in *SlZF3* overexpression lines and recovery of dwarf phenotype by exogenous GA3

**Figure 3:**
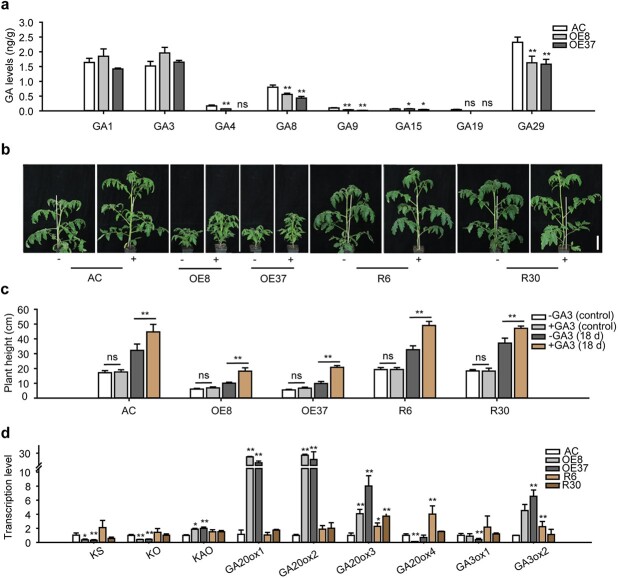
*SlZF3* regulates tomato dwarfism through the GA pathway. **a** Content of GA compounds in *SlZF3*-overexpressing lines (OE8 and OE37) and wild-type (AC). The shoot apex of 1-month-old seedlings was used for GA extraction and analysis. Values are mean ± standard deviation, Student’s *t*-test (*n* = 6). **b** Phenotype of overexpression lines, RNAi lines (R6, R30), and wild-type (AC) treated (+) or not treated (−) with GA3. One-month-old tomato seedlings were sprayed with either 100 μM exogenous GA3 or water six times at 3-day intervals. Scale bar = 10 μm. **c** Height of plants shown in (**b**). Values are mean ± standard deviation, Student’s *t*-test (*n* = 9). ^**^P < .01; ns, no significant difference. **d** Transcription level of genes in the GA biosynthesis pathway detected by RT–qPCR. The tomato *ACTIN* gene was used as the internal control. Values are mean ± standard deviation, Student’s *t*-test (*n* = 3). ^*^P < .05. ^**^P < .01; ns, no significant difference.

Many studies have shown that GA is closely involved in the regulation of plant height and leaf expansion [9, 12, 53]. Besides, the expression profile of *SlZF3* showed that it is inducible by GA3 (Fig. 1b). Therefore, we examined whether the GA level is changed in dwarf plants caused by *SlZF3* overexpression. Results of liquid chromatography–mass spectroscopy (LC–MS) showed that the contents of GA4, GA8, GA9, and GA29 were significantly reduced in the *SlZF3* overexpression lines compared with AC (Fig. 3a). This indicated that *SlZF3* regulates tomato development through the GA pathway.

To test this, we checked whether exogenous GA can rescue the dwarf phenotype of the overexpression lines. With 100 μM exogenous GA treatment, it was shown that the height of the *SlZF3* overexpression plants largely recovered to that of wild-type (Fig. 3b and c), suggesting that *SlZF3* regulates tomato plant height through the GA synthesis pathway rather than GA signaling.

### Altered expression of genes in GA pathway of *SlZF3* transgenic lines

To identify the potential target genes of SlZF3, we further examined the transcription level of representative genes involved in the GA biosynthesis pathway. It was found that the transcripts of *KS*, *KO*, *SlGA20ox4*, and *SlGA3ox1* decreased significantly in the overexpression lines, while the transcripts of *KAO*, *SlGA20ox1*, *SlGA20ox2*, *SlGA20ox3*, and *SlGA3ox2* increased significantly (Fig. 3d). Among the four GA oxidase genes, only *SlGA20ox4* showed decreased expression, and the opposite expression pattern was also shown in the RNAi lines. The decrease in its expression was consistent with the result that the level of GA4 in *SlZF3* overexpression lines was lower than that of AC (Fig. 3a). Besides, SlZF3 is highly likely to act as a transcription repressor because it has an ERF-associated amphiphilic repression (EAR) motif in its C-terminus [50], which is also consistent with the downregulation of *SlGA20ox4* in the overexpression lines. In addition, previous studies also supported the idea that the downregulation of GA oxidase leads to plant dwarfism [7, 54]. We hypothesized that *SlGA20ox4* could be the target of SlZF3, although we cannot rule out that the upstream *SlKS* and *SlKO* can also be putative targets.

### SlZF3 suppressed *SlGA20ox4* expression by promoter binding

**Figure 4:**
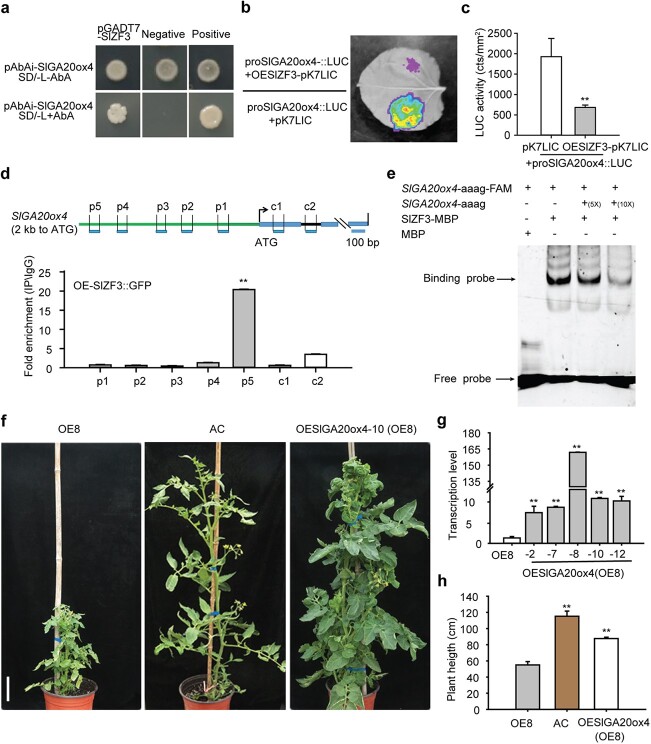
SlZF3 binds to the *SlGA20ox4* promoter and suppresses its expression. **a** Y1H assay. Bait (pAbAi-SlGA20ox4) and prey (pGADT7-SlZF3) vectors were co-transformed into yeast strain Y1HGold and plated on SD/−Leu−Ura medium with or without AbA (30 ng/ml). pAbAi-SlGA20ox4 + pGADT7 and pGADT-Rec2–53 were used as negative and positive control, respectively. **b** Luciferase-based transactivation assay. proSlGA20ox4::LUC and OESlZF3-pK7LIC were co-infiltrated into *N. benthamiana* leaves before fluorescence signal detection. proSlGA20ox4::LUC + pK7LIC was used as the control. **c** Luciferase activity in assay shown in (**b**). Values are mean ± standard deviation, Student’s *t*-test (*n* = 6). **d** ChIP–qPCR result. The *SlGA20ox4 *promoter was divided into five fragments (p1–p5) from the start codon to upstream 2000 bp. c1 and c2 are control fragments located in the exon and intron of* SlGA20ox4*, respectively. ChIP–qPCR was performed to quantify the relative enrichment of each fragment. The data represent means and standard deviation of two biological replicates (Student’s *t*-test). **e** EMSA. *SlG20ox4*-AAAG-FAM represents the probe fragment of *SlG20ox4* promoter containing DOFCOREZM element (AAAG). *SlG20ox4*-AAAG represents the competition probe. + and – indicate presence and absence, respectively. 5× and 10× indicate the relative concentration of competition probe. **f** Plant height recovery of *SlZF3* overexpression line (OE8) by overexpressing *SlGA20ox4*. Scale bar = 10 cm. **g**, **h** Transcription level of *SlGA20ox4* (**g**) and height (**h**) of OE8 plants overexpressing *SlGA20ox4*. Values are mean ± standard deviation, Student’s *t*-test (*n* = 5). ^**^P < .01.

To test our hypothesis, we performed a series of biochemical experiments to study the interaction of SlZF3 and the promoter of *SlGA20ox4.* First, a yeast one-hybrid (Y1H) assay was conducted to check whether SlZF3 can bind to the promoter of *SlGA20ox4*. Yeast cells co-transformed with AD-SlZF3 and pAbAi-ProSlGA20ox4, as well as the positive control, could grow in selective medium (SD−Leu−Ura) containing aureobasidin A (AbA), but the negative control did not show growth (Fig. 4a). This result indicated that SlZF3 can interact with the *SlGA20ox4* promoter in yeast cells. Then, a luciferase assay was performed to test whether SlZF3 can affect the expression of *SlGA20ox4 in vivo*. When OEZF3-pK7LIC and proSlGA20ox4::LUC were co-infiltrated into tobacco (*Nicotiana benthamiana*) leaves, the fluorescence signal was significantly decreased compared with the control treatment co-infiltrated with the control effect vector (pK7LIC) and proSlGA20ox4::LUC (Fig. 4b and c). This result indicated that SlZF3 can suppress the transcription of *SlGA20ox4* by binding to its promoter. Further, a ChIP (chromatin immunoprecipitation)–qPCR was carried out to verify the interaction of SlZF3 and the *SlGA20ox4* promoter*.* It turned out that SlZF3-GFP fusion protein can bind to the p5 region (−1816 to −1912 bp) of the *SlGA20ox4* promoter (Fig. 4d). The qPCR signal from p5 was >10 times than those from other promoter regions and the control (exon and intron region). In order to identify the potential regulatory sequence in the p5 region of the *SlGA20ox4* promoter that SlZF3 binds to, we conducted reverse Y1H screening. Six positive clones were identified and sequenced. Five known *cis*-elements (ANAERO2CONSENSUS, ARR1AT, CAATBOX1, BOXLCOREDCPAL, and DOFCOREZM) were predicted using the online New PLACE tool, among which the CAATBOX1 element was detected in two of the positive clones (Supplementary Data Table **S4**). The DOFCOREZM element (AAAG) was identified in the p5 sequence in our ChIP–qPCR experiment, suggesting that SlZF3 can bind to this element. To confirm this, we performed an electrophoretic mobility shift assay (EMSA) to check whether SlZF3 can bind the probe containing the DOFCOREZM element (Fig. 4e). It was shown that SlZF3 protein could indeed bind to the probe with the DOFCOREZM element and caused a mobility shift, while the competition probe weakened the signal of the shifted band (Fig. 4e). All these results verified that SlZF3 can regulate *SlGA20ox4* by direct promoter binding.

Finally, we examined the genetic interaction of *SlZF3* and *SlGA20ox4* by overexpressing *SlGA20ox4* in the *SlZF3* overexpression line OE8. It was found that the *SlGA20ox4*-overexpressing plants with the OE8 background showed the same plant height as the wild-type (Fig. 4f–h). This further confirmed that *SlGA20ox4* is an important target gene of SlZF3, which is suppressed by SlZF3 through promoter binding.

## Discussion

### Overexpression of *SlZF3* results in GA deficiency and semi-dwarf phenotype

Height is one of the most important agronomic traits for crops, and affects plant architecture, soil utilization rate, nutrition management, and production management [1, 55]. In this study, we found that the C2H2 zinc-finger transcription factor SlZF3 regulates tomato plant height via the GA pathway.

*SlZF3* overexpression lines displayed a semi-dwarf phenotype, whereas its RNAi lines were taller than the wide-type AC (Fig. 2a). Numerous studies show that either internode number or internode length is the determinant of plant height [27, 29]. Our results showed that SlZF3 affects plant height through the length of internodes rather than the number of internodes (Fig. 2d, Supplementary Data Table **S1**). Further investigation revealed that SlZF3 regulates internode length via cell elongation (Fig. 2e and f), which is a typical effect of gibberellin [56]. Nevertheless, SlZF3 not only affects cell length, but also may affect cell division. We calculated the number of cells in the fourth internode based on internode and cell length. Approximately 210 and 170 cells were estimated for lines R6 and R30, respectively, while the corresponding number for wild-type was ~300. This may explain the non-obvious difference in internode length between the RNAi lines and wild-type. Defects of enzymes in the GA biosynthesis pathway cause shortened plants and internodes, and defects in early-step enzymes can even lead to serious dwarfism and stunted growth [18, 19]. Meanwhile, the defects in the GA biosynthesis pathway are usually rescuable by exogenous GA [24, 27].

In agreement with this, the GA compounds, such as GA4, GA8, GA9, GA19, and GA29, were significantly reduced in *SlZF3* overexpression lines compared with the wild-type (Fig. 3c). Previous studies have found that a decrease in GA4 (a bioactive gibberellin) will lead to dwarfing of the plant [10]. Both GA9 and GA19 can be produced by GA20ox, which is an important oxidase in GA biosynthesis [11]. Besides, the levels of GA9 and GA19 in the *SlZF3* overexpression lines also decreased, which can lead to a decrease in bioactive GA4 in the last step. Therefore, reduced GA compounds could be the reason for the dwarfism of *SlZF3* overexpression lines. We further applied exogenous GA3 to test whether it can rescue the dwarf phenotype of the overexpression lines. The results showed that exogenous GA indeed recovered the dwarfing phenotype (Fig. 3b and c). As exogenous GA3 could not rescue the plant defects in the GA signaling pathway [53, 57]. We speculated that *SlZF3* can regulate tomato plant height through the GA biosynthesis pathway. To identify the target gene of SlZF3, we first detected the transcript of GA biosynthesis genes in the transgenic lines by RT–qPCR. Because SlZF3 contains an EAR motif at the C-terminus [50], and the overexpression lines have a typical GA deficiency phenotype, we focused our attention on GA biosynthesis genes with reduced expression in *SlZF3* overexpression lines but increased expression in RNAi lines. This led us to regard *SlGA20ox4* as our first choice. GA20 oxidase is responsible for the synthesis of GA9 and GA19 in the pathway of GA biosynthesis [11]. Our previous study also found that suppression of *SlGA20ox2* caused the dwarf phenotype in tomato, while plants with a reduced *SlGA20ox3* transcription level showed no visible changes in stems [54]. Nevertheless, no functional study has been performed on *SlGA20ox4*. In brief, the semi-dwarf phenotype of the *SlZF3* overexpression lines resulted from GA deficiency, which was likely caused by the repression activity of SlZF3 on GA biosynthesis genes, such as *SlGA20ox4*.

### SlZF3 regulates tomato plant height by directly binding to the promoter of GA genes

A variety of biochemical experiments have been conducted to study the interaction of SlZF3 and the *SlGA20ox4* promoter. The Y1H result showed that SlZF3 interacts with *SlGA20ox4* promoter in yeast cells (Fig. 4a). The transactivation assay based on the luciferase reporter further showed that SlZF3 can repress the promoter activity of *SlGA20ox4* in tobacco leaves (Fig. 4b and c)*.* Further, the ChIP–qPCR result showed that SlZF3-GFP fusion protein can bind to the specific region of the *SlGA20ox4* promoter (Fig. 4d). In addition, several putative *cis*-elements that SlZF3 may bind were identified by reverse Y1H, and EMSA confirmed that SlZF3 can bind to the probe of the *SlGA20ox4* promoter containing the DOFCOREZM element (Supplementary Data Table **S4** and Fig. 4e). Finally, we performed a genetic assay by overexpressing *SlGA20ox4* in one of the *SlZF3* overexpression lines (OE8), and it was found that *SlGA20ox4* overexpression can recover the plant height of OE8 (Fig. 4f and h). All these results support the idea that SlZF3 can directly bind the promoter of the GA biosynthesis gene *SlGA20ox4*, resulting in the repression of *SlGA20ox4* and consequently repressing the growth of tomato plants.

In addition to *SlGA20ox4*, other genes in the GA pathway could also be the targets of SlZF3. The transcripts of *SlKS* and *SlKO* were also lower in the *SlZF3* overexpression lines, although there was no significant difference in the RNAi lines (Fig. 3d). We also performed Y1H and luciferase assays on SlZF3 and the promoter of these two genes. Similar to *SlGA20ox4*, it was found that SlZF3 can bind to the promoter of *SlKS* and *SlKO* in yeast cells (Supplementary Data Fig. **S2**) and the luciferase assays showed that SlZF3 represses the promoter activity of *SlKS* and *SlKO* in tobacco leaves (Supplementary Data Fig. **S3**). However, previous reports showed that severe dwarfism can be caused by defects of upstream genes in the GA biosynthesis pathway [58–60]. We speculated that the degree of dwarfism depends on the level of target gene inhibition and the final reduced dose of GA.

Other transcription factors have been documented to regulate plant height through the GA pathway. For example, OsGRF7 can regulate GA and IAA pathways by binding the ACRGDA motif in the promoter of a cytochrome P450 gene (*OsCYP714B1*) and *OsARF12*, thus shaping plant architecture in rice [61]. Overexpression of *CmDRP* (a YAB transcription factor) resulted in a semi-dwarf phenotype with reduced bioactive GA3 level. CmDRP can directly bind to the *CmGA3ox1* promoter and inhibit its expression in chrysanthemum [62]. HY5 as a master regulator is involved in many aspects of plant development and defense [63]. It has been shown that HY5 targets many of the GA metabolism genes [64]. The HY5 ortholog in pea, LONG1, negatively regulates GA levels by inducing the expression of *GA2ox2*, a key GA catabolic gene [65]. As far as we know, this is the first report of regulation of plant height by a C2H2 zinc finger through direct binding to GA pathway genes.

### SlZF3 plays an important role in coordinating tomato development and stress tolerance

**Figure 5:**
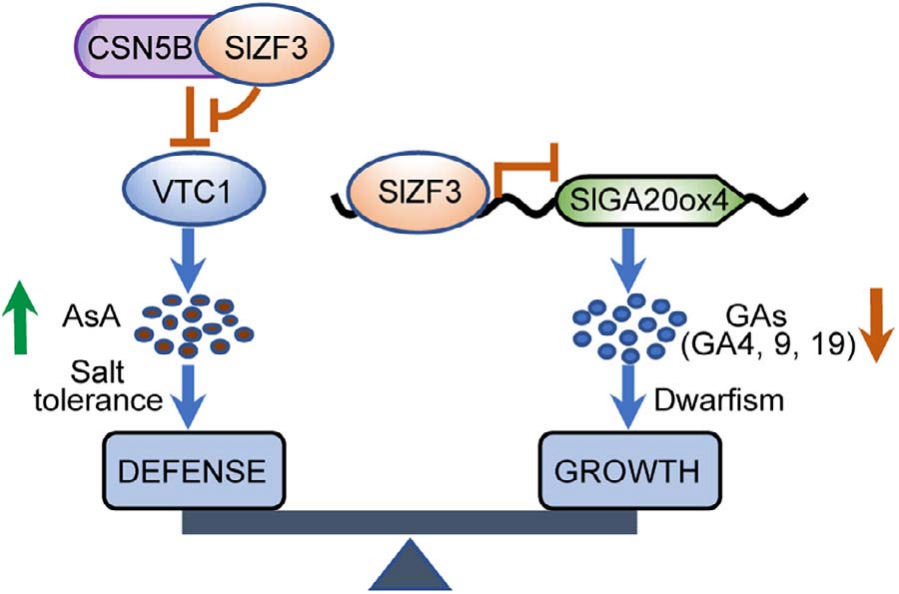
SlZF3 coordinates plant development and defense in tomato. SlZF3 can directly bind to the promoter of GA biosynthesis genes such as *SlGA20ox4* to repress their transcription, which leads to a reduced level of bioactive GAs and dwarfism of plants. Our previous work showed that SlZF3 can physically interact with CSN5B, inhibiting the degradation of VTC1 by CSN5B, and consequently promotes the biosynthesis of ascorbic acid (AsA) and enhances plant defense (salt tolerance) by scavenging ROS [50].

The function of a transcription factor can be versatile, and SlZF3 could be such an important regulator. Our previous study has revealed that SlZF3 enhances plant salt-stress tolerance by interacting with SlCSN5B, a subunit of the COP9 signalosome, to promote VTC1-mediated ascorbic acid biosynthesis and thus ROS scavenging capability [50]. By combining the biochemical and genetic evidence revealed in this study, we proposed a working model for the coordinating role of SlZF3 in tomato growth and defense (Fig. 5). Overexpression of *SlZF3* represses tomato plant growth via the GA pathway by directly binding to the promoter of GA biosynthesis genes (such as *SlGA20ox4*, *SlKS*, and *SlKO*) and inhibiting transcription. We demonstrated that SlZF3 can bind to a probe containing the DOFCOREZM element (AAAG) in the promoter of *SlGA20ox4*. Repression of the GA pathway results in decreases in GA compounds (such as GA4, GA9, and GA19), leading to the dwarfing phenotype in tomato. Meanwhile, the overexpression of *SlZF3* enhances the ascorbic acid pathway and ROS scavenging ability of transgenic plants. Therefore, our study shows that SlZF3 acts as an important coordinator in tomato growth and abiotic stress tolerance.

Other zinc finger genes have also been demonstrated to participate in multiple biological processes. OsDRZ1, for example, enhances both drought tolerance and plant growth in rice [66]. Overexpression of a CCCH-tandem zinc finger protein, OsTZF5, increases rice drought resistance, but simultaneously inhibits plant growth. The negative impact of *OsTZF5* on growth can be overcome by employing the stress-responsive promoter of *OsNAC6* [67]. A similar strategy can also be applied to *SlZF3*. SlZF3 might regulate genes with the promoter harboring specific *cis*-elements, such as ANAERO2CONSENSUS (AGCAGC) identified in our reverse Y1H analysis (Supplementary Data Table **S4**), which is involved in anaerobic stress [68]. Besides, it was reported that the R2R3 MYB transcription factor DcMYB1 can bind to the element BOXLCOREDCPAL (ACCWWCC) in the *DcPAL1* promoter, and upregulate its expression under UV-B light [69]. This element is also a potential target of SlZF3 (Supplementary Data Table **S4**).

There are cases in which transcription factors are reported to be involved in several pathways to balance plant development and environmental adaptation. It has been reported that ethylene-responsive factors can regulate different pathways in plants. For example, *AtERF019*-overexpressing *Arabidopsis* plants show delayed flowering and senescence, while these plants also show increased tolerance to water deficiency, suggesting that *AtERF019* plays dual roles in plant growth and drought tolerance [70]. In rice, *OsERF83*-overexpressing lines display higher photochemical efficiency and increased drought tolerance. However, the overexpression of *OsERF83 *leads to growth retardation and reduced grain yield [71]. Other types of factor, such as the small heat shock protein AsHSP26.8, can coordinate the growth, development and stress response of the plant. *AsHSP26.8* overexpression lines show twisted leaf blades and a slow growth rate but improved drought and salt tolerance in creeping bentgrass [72]. The MADS-box transcription factor SlMBP22 can participate in the regulation of tomato growth and drought tolerance [73]. Our previous and present work support the idea that SlZF3 coordinates plant development and stress tolerance in tomato.

## Materials and methods

### Plant materials, growth conditions, and treatments

Transgenic lines with overexpression (OE8 and OE37) or knockdown (RNAi lines, R6 and R30) of *SlZF3* (Solyc06g075780) on the AC genetic background were used in this study [50]. We also generated transgenic lines for chromatin immunoprecipitation (ChIP) (OE-SlZF3::GFP) on the AC background, and for overexpression of *SlGA20ox4* on the genetic background of OE8 (OESlGA20ox4(OE8)). All plants were grown in a growth room, with room temperature (25 ± 2°C) and a 16 hours light/8 hours dark cycle.

To investigate the tissue expression profile of *SlZF3*, different tissues (roots, stems, leaves, flowers) were collected from 9-week-old seedlings, and fruits at different developmental stages were collected from adult plants. Three biological replicates with three plants per replicate were prepared.

To explore the expression pattern of *SlZF3* under different hormone treatments, 6-week-old AC seedlings were treated with various hormones by spray assay [74], including 100 μM gibberellic acid (GA3), 1% (v/v) ETH, 100 μM IAA, and 100 μM ABA, with water as control. The third leaf from the top was collected for RNA isolation, with three biological replicates and three plants per replicate.

To test whether exogenous GA3 can recover the dwarf phenotype of *SlZF3*-overexpressing lines, 1-month-old seedlings of the overexpression lines (OE8, OE37) and RNAi lines (R6, R30), together with the wild-type (AC), were sprayed six consecutive times with 100 μM GA3 (in 0.02% Tween-20) at intervals of 3 days. Plants sprayed with an equal volume of the solvent (0.02% Tween-20 in water) were set as the control group. After spraying, plant height was measured according to the standard mentioned below.

### Phenotyping of the transgenic plants

To characterize the dwarf phenotype, plant height, internode number, internode length, and stem cell length were measured for 1-month-old seedlings of all the lines (OE8, OE37, R6, R30, and AC). Nine plants were recorded for each line tested. For plant height, the height of the plant was measured as the distance from the soil surface to the shoot apex. To count the number of internodes, the first internode was designated as the internode between the cotyledons and the first true leaf of the main stem. The number of internodes was recorded from the cotyledon to the top of the main stem. The internode length of each internode was also measured. To measure the length of stem cells, the middle part of the fourth internode (from the bottom) was selected and stained with 100 μg/ml propidium iodide (PI) (Sigma, USA) for 30 minutes. After washing with distilled water three times, the samples were imaged with a Leica SP8 confocal microscope (Leica, Germany), and the excitation wavelength was 488 nm. The cell length of all samples was measured using the software Image J (https://imagej.nih.gov/ij/)http://rsb.info.nih.gov/ij/. To investigate the height of *SlGA20ox4*-overexpressing plants, we could only use *T*_0_ plants because of no seed production by the transgenic plants. Well-rooted regenerated plants of OE8, AC, and OESlGA20ox4(OE8) were transplanted to 20-cm-diameter pots. Measurement of plant height was performed on five plants for each material 60 days post-transplanting.

The leaf phenotypes were also investigated, including the length, width, and thickness of the third leaf from the bottom. To characterize the leaf thickness, we made paraffin sections of leaves with five biological replicates and three leaf pieces per replicate. Formaldehyde–acetic acid–ethanol (FAA) fixative solution was used to fix all the samples, and paraffin sections were prepared according to a published protocol [75]. Leaf thickness was also measured using the software ImageJ.

### Measurement of gibberellin content

To measure the different compounds of gibberellin in *SlZF3*-overexpressing lines, shoot apexes were collected from OE8 and OE37 seedlings (1 month old). The samples were sent to MetWare (http://www.metware.cn/) for gibberellin measurement using an LC–MS/MS method according to a published protocol [76].

### Generation of transgenic lines

To generate transgenic lines for ChIP analysis, the full-length coding sequence of *SlZF3* (Solyc06g075780) without the stop codon was amplified by Phanta Max Super-Fidelity DNA Polymerase (Vazyme, China) (primers are listed in Supplementary Data Table S2). The PCR product was cloned into pENTR™/SD/D-TOPO vector (Invitrogen, Carlsbad, CA, USA) via the TOPO cloning method, and then recombined to the gateway vector pGWB451 [77] using the LR recombination reaction according to the supplier’s instructions. The resulting vector was introduced into GV3101 (strain of *Agrobacterium tumefaciens*) by electroporation and then transformed into tomato AC using the leaf disc method [78].

To overexpress *SlGA20ox4* (Solyc01g093980) on the background of the *SlZF3* overexpression line OE8 (with kanamycin resistance), an expression vector was firstly constructed with a hygromycin (Hrg) resistance marker. The *hptII* (hygromycin resistance) gene together with its promoter and terminator was amplified from the vector pCAMBIA1302 by DNA polymerase, using the primer pair P35S-G8Hrg-F and P35S-G8Hrg-R (Supplementary Data Table **S2**). The PCR product was digested with *Mlu*I and *Sac*I and inserted into *Mlu*I/*Sac*I linearized plant expression vector pHELLSGATE8 [79] using T4 DNA Ligase (NEB, Beverly, MA, USA). The resulting vector was designated pHELLSGATE8-Hrg*.* The coding sequence of *SlGA20ox4* was amplified with the primer pair OESlGA20ox4-F and OESlGA20ox4-R (Supplementary Data Table **S2**) and recombined into pHELLSGATE8-Hrg by using Exnase II (Vazyme, China). The resulting vector was introduced into GV3101 and then into OE8 as described [78].

### Reverse transcription polymerase chain reaction

For gene expression analysis, total RNA of the above-mentioned tomato samples was extracted by using TRIzol reagent (Invitrogen). The HiScript^®^ II 1st Strand cDNA Synthesis Kit (Vazyme) was used to synthesize cDNA. Primers were designed using online Primer3 (http://bioinfo.ut.ee/primer3-0.4.0/primer3) (Supplementary Data Table **S3**). SYBR Green-based RT–qPCR was performed in the LightCycler 480 system (Roche Applied Science, Mannheim, Germany) according to an established program [50]. *ACTIN* (Solyc11g005330) was used as the internal control. The relative expression level was calculated by the ΔΔCt method [80].

### Yeast one-hybrid assay

The cDNA of *SlZF3* was amplified and cloned into pGADT7 to obtain the prey vector (AD-SlZF3). A 2-kb promoter fragment of *SlGA20ox4*, *SlKS* (Solyc07g066670), and *SlKO* (Solyc04g083160) was amplified by specific primers (Supplementary Data Table **S2**). The amplified promoter was recombined into *pAbAi*, and the resulting bait vectors were designated pAbAi-SlGA20ox4, pAbAi-SlKS, and pAbAi-SlKO, respectively. The bait vector was transformed into yeast strain Y1HGold following the LiAc/SS-DNA/PEG transformation method (Clontech, Mountain View, CA, USA) and plated onto nutrient-deficient SD−Ura medium. Subsequently, the prey vector was transformed into the yeast strain Y1HGold containing the bait vectors, and cultured on SD−Leu−Ura medium. The yeast culture with the positive clone was diluted to OD600 = 0.1 with 0.9% NaCl, and the diluted suspension was spotted on SD−Leu−Ura medium with or without AbA.

### Reverse yeast one-hybrid assay

To identify the putative *cis*-regulatory elements that SlZF3 binds to, a reverse Y1H assay was performed. The cDNA of *SlZF3* was amplified with primers Rec2-SlZF3-F and Rec2-SlZF3-R (Supplementary Data Table **S2**) and cloned into pGADT7-Rec2 to obtain the bait vector (Rec2-SlZF3). Rec2-SlZF3 was transformed into yeast strain Y187 with the random DNA library [81] and plated on SD−His−Leu−Trp medium containing 30 mM 3-AT (3-amino-1,2,4-triazole, Thermo, USA). Positive clones were identified, cultured, and sequenced. The online New PLACE tool (https://www.dna.affrc.go.jp/PLACE/?action=newplace) was used to predict potential *cis*-elements that SlZF3 may bind to.

### Transactivation assay

To test whether SlZF3 can regulate the expression of putative target genes (*SlGA20ox4*, *SlKS*, and *SlKO*) in the GA biosynthesis pathway, transactivation assays were performed using luciferase as the reporter driven by the promoters of these genes. The cDNA of *SlZF3* was amplified using OEZF3-LIC-F and OEZF3-LIC-R (Supplementary Data Table **S2**) and inserted into pK7LIC vector [82] using Exnase II recombinase (Vazyme). The resulting effect vector was designated OEZF3-pK7LIC. The 2-kb promoter fragment of *SlGA20ox4*, *SlKS*, and *SlKO* was amplified by corresponding primers (Supplementary Data Table **S2**) and cloned into the luciferase reporter vector pHELLSGATE8-LUC. The vector pHELLSGATE8-LUC was constructed by insertion of the luciferase reporter gene into pHELLSGATE8. The luciferase gene was amplified from pGreenII 0800 [83] using primers GATE8-LUC-F and GATE8-LUC-R (Supplementary Data Table **S2**), and combined into pHELLSGATE8 linearized by *Xba*I and *Xho*I. All these vectors were introduced into GV3101. The *Agrobacterium* strain carrying the reporter vector was mixed equally with either the effect vector (OEZF3-pK7LIC) or the negative control vector (pK7LIC), and co-injected into the lower side of tobacco (*N. benthamiana*) leaves. The infiltrated tobacco plants were grown overnight under darkness and moved to normal growth condition. Three days later, the infiltrated leaves were spread with 50 mg/L d-luciferin (Promega, Madison, USA). The luciferase fluorescence was detected by a NightSHADE LB985 imaging system (Berthold Technologies, Bad Wildbad, Germany). The fluorescence signal was measured using the IndiGO^TM^ software (Berthold Technologies).

### Chromatin immunoprecipitation–qPCR

The ChIP–qPCR experiment was performed using transgenic plants overexpressing the SlZF3::GFP fusion protein, according to published methods [84, 85]. Briefly, young leaves from transgenic tomato plants were sliced and cross-linked for 30 minutes in a cross-linking buffer (10 mM MgCl_2_, 10 mM Tris–HCl, pH 8.0 and 0.44 M sucrose) containing 1% formaldehyde under vacuum. After crosslinking was terminated with glycine, the samples were cleaned with sterilized ddH_2_O and ground into powder in liquid nitrogen. Honda Buffer (Roche, USA) was used for nuclear extraction, then Diagenode Bioruptor (Denville, NJ, USA) was used for fragmentation of chromatin into fragments of ~500 bp. The sonicated chromatin was incubated with anti-GFP (Abcam, Cambridge, USA) and protein A Dynabeads (Invitrogen) at 4°C overnight. After purifying the DNA released from antibody–chromatin complexes, the released DNA was used as the template for qPCR analysis. The 2-kb promoter of *SlGA20ox4* was divided into five fragments (Amp 1, 2, 3, 4, and 5) based on their location in the promoter, starting from the start codon. Meanwhile, two fragments located in the first exon and first intron of *SlGA20ox4* were used as the control templates (CK1 and CK2). All the primers for ChIP–qPCR analysis are listed in Supplementary Data Table **S3**. The qPCR program was the same as that for RT–qPCR.

### Electrophoretic mobility shift assay

To test whether SlZF3 can bind to the *SlGA20ox4* promoter via the DOFCOREZM element, an EMSA assay was performed. The cDNA of *SlZF3* was amplified using SlZF3-MBP-F and SlZF3-MBP-R (Supplementary Data Table **S2**) and inserted into pMAL-c2X [86] containing the maltose-binding protein (MBP) gene using Exnase II recombinase (Vazyme). The resulting vector was transformed into *Escherichia coli* strain BL21(DE3) to express an MBP-tagged SlZF3 protein (SlZF3-MBP). Based on the sequence of the p5 region of the *SlGA20ox4* promoter, a unique 36-bp single-stranded oligo containing the DOFCOREZM element (AAAG) was synthesized and labeled with FAM at its 5′-end (Sangon Biotech, Shanghai, China). The same sequence without FAM label served as the competition probe. The EMSA assay was performed essentially as described [87]. Purified SlZF3-MBP protein was incubated with the probe for 30 minutes. The DNA–protein complex was separated by a 6% native PAGE (polyacrylamide gel electrophoresis) gel using 0.5 × TBE (Tris–borate–EDTA) buffer. After 1 hour of electrophoresis, the gel image was captured using a FluorChem M system (ProteinSimple, San Jose, USA).

## Supplementary Material

Web_Material_uhad025

## Data Availability

All relevant data can be found within the manuscript and its supporting materials. The sequence data were downloaded from the Sol Genomics Network (https://www.sgn.cornell.edu/)https://solgenomics.net/ database with the following accession numbers: *SlZF3*, Solyc06g075780; *SlGA20ox1*, Solyc03g006880; *SlGA20ox2*, Solyc06g035530; *SlGA20ox3*, Solyc11g072310; *SlGA20ox4*, Solyc01g093980; *SlK*S, Solyc07g066670; *SlKO*, Solyc04g083160; *SlKAO*, Solyc01g080900; *Actin*, Solyc11g005330; *GA3ox1*, Solyc06g066820; *GA3ox2*, Solyc03g119910.
