# Supplementary material for: SlZF3 regulates tomato plant height by directly repressing SlGA20ox4 in the gibberellic acid biosynthesis pathway
Source: Hortic Res. 2023 Feb 21;10(4):uhad025. doi: 10.1093/hr/uhad025 (PMC10116951; doi:10.1093/hr/uhad025)
Supplement: Web_Material_uhad025 [file web_material_uhad025.zip › HR-2022-1005R1-Supplementary.docx]

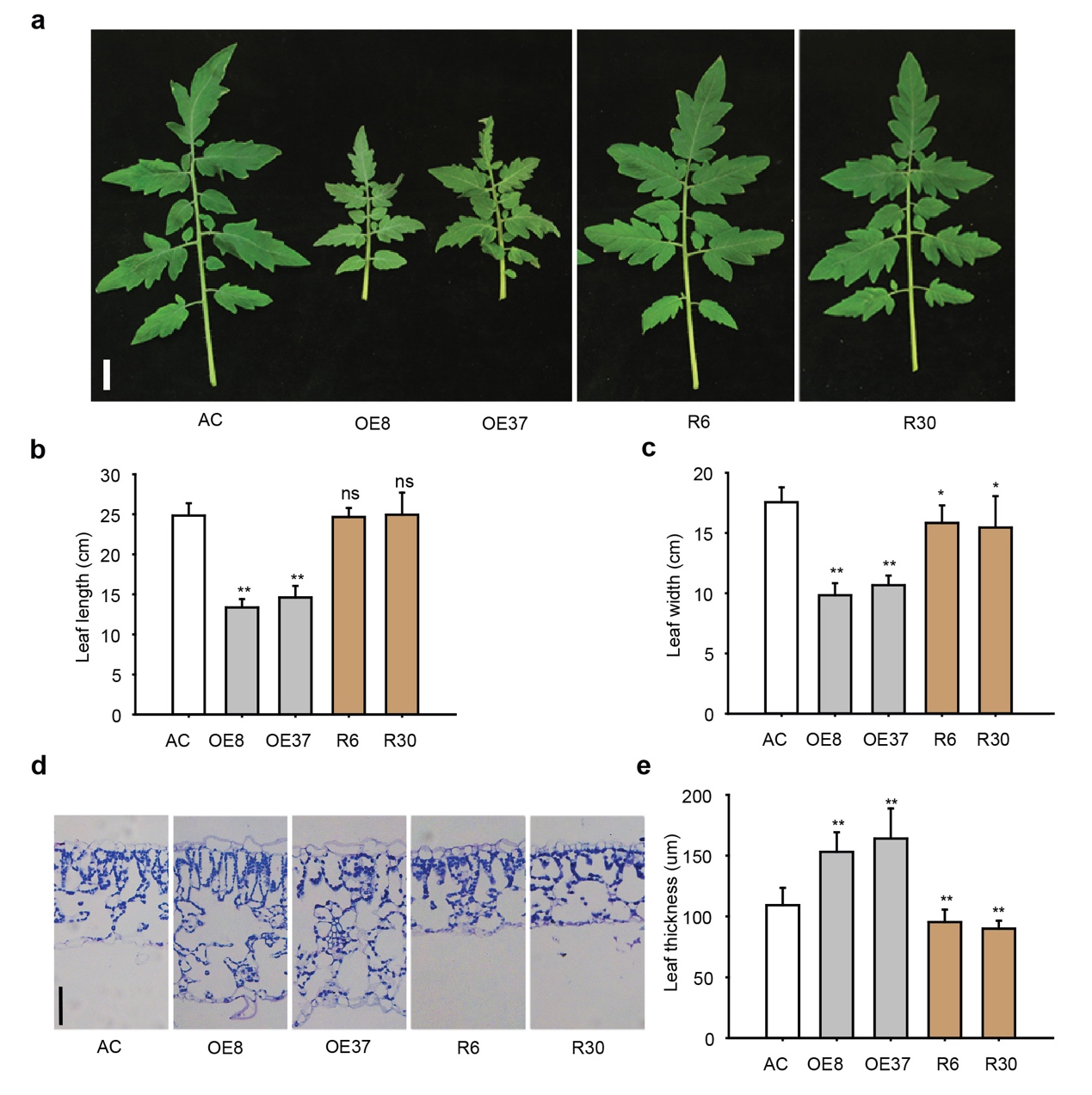


**Figure S1 Overexpression of *SlZF3* affects leaf** **expansion and leaf thickness.**

**a**, Leaf phenotypes of the overexpression (OE8, OE37) and RNAi (R6, R30) lines of *SlZF3* in Alisa Craig (AC) background. The third leaf from the top was collected from six-week-old plants. Bar = 2 cm. **b**-**c**, Length (**b**), width (**c**), and paraffin sections (**d**) and thickness (**e**) of the leaves in (**a**). Values are mean ± SD, Student’s t-test (n = 9). Bar = 50 μm (**d**). Student’s t-test, * P<0.05. ** P<0.01. ns, no significant difference.


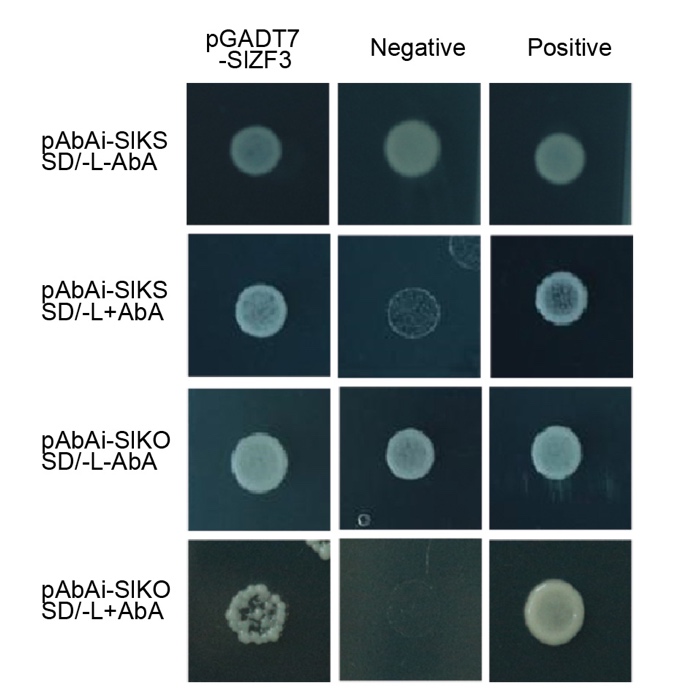


**Figure S2 SlZF3 binds to the promoter of *SlKS and SlKO* in a yeast cells.**

The bait (pAbAi-SlKS or pAbAi-SlKO) and prey (pGADT7-SlZF3) vectors were co-transformed into the yeast strain Y1HGold and plated on SD-Leu-Ura medium with or without Aureobasidin A (15 ng/ml). pAbAi-SlKS/pAbAi-SlKO + pGADT7/pGADT-Rec2-53 were used as negative or positive control, respectively.


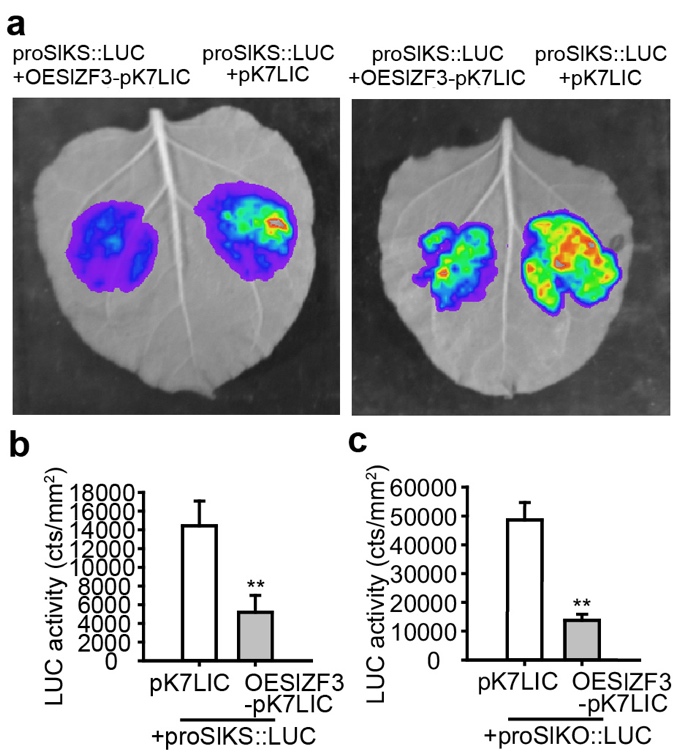


**Figure S3 SlZF3 binds to the promoter of *SlKS and SlKO* *in vivo*.**

**a**, Luciferase-based transactivation assays in *N. benthamiana* leaves. OESlZF3:pK7LIC and proSlKS::LUC or proSlKO::LUC were co-infiltrated in tobacco leaves before fluorescence signal detection. pK7LIC + proSlKS::LUC/proSlKO::LUC was used as the control. **b**, Luciferase activity of (**a**). Values are mean ± SD, Student’s t-test (n = 6). ** P<0.01.
