# Supplementary material for: SlZF3 regulates tomato plant height by directly repressing SlGA20ox4 in the gibberellic acid biosynthesis pathway
Source: Hortic Res. 2023 Feb 21;10(4):uhad025. doi: 10.1093/hr/uhad025 (PMC10116951; doi:10.1093/hr/uhad025)
Supplement: Web_Material_uhad025 [file web_material_uhad025.zip › Luo MS-Table S1.docx]

**Supplementary Data**

**Table S1** The internode length of the SlZF3 overexpression and RNAi lines.

| Line | Internode | | | | | |
| --- | --- | --- | --- | --- | --- | --- |
|  | 1st | 2nd | 3rd | 4th | 5th | 6th |
| AC | 1.1±0.3ab | 2.0±0.9b | 2.2±0.7a | 3.6±0.7a | 2.6±0.8a | 1.4±0.5a |
| OE8 | 0.3±0.1c | 0.5±0.1c | 0.6±0.2b | 0.7±0.2b | 0.6±0.2b | 0.4±0.2b |
| OE37 | 0.3±0.1c | 0.5±0.2c | 0.7±0.2b | 0.7±0.1b | 0.5±0.1b | 0.4±0.1b |
| R6 | 1.5±0.6ab | 2.9±0.7ab | 2.1±1.0a | 4.0±1.0a | 2.7±0.8a | 1.4±0.5a |
| R30 | 1.1±0.5b | 2.3±1.0ab | 2.9±1.5a | 3.4±0.7a | 2.5±0.7a | 1.5±0.7a |

Note: The data are shown as mean ± SD (n =9), and submitted to one-way analysis of variance (ANOVA) and Duncan’s multiple range test. A column followed by the same letter are not significantly different at p<0.05.
