# Supplementary material for: SlZF3 regulates tomato plant height by directly repressing SlGA20ox4 in the gibberellic acid biosynthesis pathway
Source: Hortic Res. 2023 Feb 21;10(4):uhad025. doi: 10.1093/hr/uhad025 (PMC10116951; doi:10.1093/hr/uhad025)
Supplement: Web_Material_uhad025 [file web_material_uhad025.zip › Luo MS-Table S2.docx]

**Supplementary Data**

**Table S2** List of primers used in this study.

| Primer name | Primer sequence 5’-3’ |
| --- | --- |
| GA20ox4-PAbai-F: | AGCTTGAATTCGAGCTCGGTACCCTTCCCCTCCCTGTGAG |
| GA20ox4-PAbai-R: | ACATACAGAGCACATGCCTCGAGAAGTAGATCATGTTGTAATAAC |
| KO-PAbai-F: | AGCTTGAATTCGAGCTCGGTACCCAATTGCATAATTCGTTGGCC |
| KO-PAbai-R: | ACATACAGAGCACATGCCTCGAGAGGTGTTTGGACTGGCTAC |
| KS-PAbai-F: | AGCTTGAATTCGAGCTCGGTACCCTATTAGGTGGCCGGACAC |
| KS-PAbai-R: | ACATACAGAGCACATGCCTCGAGTTGCTTAAAGAGCAAAAGAGGAG |
| ZF3-AD-F: | ACGTACCAGATTACGCTCATATGATGATAAAAATTAGAGAAG |
| ZF3-AD-R: | TACGATTCATCTGCAGCTCGAGCAGAGGATAAGAATGGAG |
| GA20ox4-G8LUC-F: | CAACGCGTTGGGAGCTCCTAATGCTCAGTTAGGTAAG |
| GA20ox4-G8LUC-R: | CGTCTTCCATCTCGAGAAGTAGATCATGTTGTAATAAC |
| KO-LUC-F: | CAACGCGTTGGGAGCTCCAATTGCATAATTCGTTGGCC |
| KO-LUC-R: | CGTCTTCCATCTCGAGAGGTGTTTGGACTGGCTAC |
| KS-LUC-F: | CAACGCGTTGGGAGCTCCTATTAGGTGGCCGGACAC |
| KS-LUC-R: | CGTCTTCCATCTCGAGTTGCTTAAAGAGCAAAAGAGGAG |
| OEZF3-LIC-F | CACTAGTTCCAGGGCGATGATAAAAATTAGAGAAG |
| OEZF3-LIC-R | GATATCATCGACCCGACGCTAAGAGGATAAGAATGGAG |
| OEZF3-GFP-F | AAAAAGCAGGCTTAATGATAAAAATTAGAGAAGACAAGG |
| OEZF3-GFP-R | AGAAAGCTGGGTAAGAGGATAAGAATGGAGG |
| OEGA20ox4-F: | CATTTGGAGAGGACAAAGCTTATGCATGATGAAAGACAAAATGATG |
| OEGA20ox4-R:  GATE8-LUC-F  GATE8-LUC-R  P35S-G8Hpy-F  P35S-G8Hpy-R  Rec2-SlZF3-F  Rec2-SlZF3-R  SlZF3-MBP-F  SlZF3-MBP-R  SlGA20ox4-AAAG-F  SlGA20ox4-AAAG-R | CTCATTAAAGCAGGACAAGCTTCTAGGTTTTGTGTAGAAGCCAATG  CATTTGGAGAGGACACGCTCGAGATGGAAGACGCCAAAAACATAAAG  TCTCATTAAAGCAGGACTCTAGATTACACGGCGATCTTTCCGCC  TGCATCCAACGCGTTGGGAGAGGCGGTTTGCGTATTG  CCGAATTAATTCGGCGTGAGCTC  ACGTACCAGATTACGCTCATATG ATGATAAAAATTAGAGAAG  TACGATTCATCTGCAGCTCGAGCAGAGGATAAGAATGGAG  GAAGGATTTCAGAATTCGGATCCATGATAAAAATTAGAGAAGACAAGGGTG  CCAGTGCCAAGCTTGCCTGCAGCTAAGAGGATAAGAATGGAGGTACAGG  GGTAAGTAAACAAAGAGTTTGTAATTTTATTTACTC  GAGTAAATAAAATTACAAACTCTTTGTTTACTTACC |
