# Supplementary material for: SlZF3 regulates tomato plant height by directly repressing SlGA20ox4 in the gibberellic acid biosynthesis pathway
Source: Hortic Res. 2023 Feb 21;10(4):uhad025. doi: 10.1093/hr/uhad025 (PMC10116951; doi:10.1093/hr/uhad025)
Supplement: Web_Material_uhad025 [file web_material_uhad025.zip › Luo MS-Table S3.docx]

**Supplementary Data**

**Table S3** **RT-qPCR primers used in this study**.

| Primer name | Primer sequence 5’-3’ |
| --- | --- |
| KAO-QF | TGCTGGATTACGAGCTTGAAAGG |
| KAO-QR | TCCCAAGCAGTTGTCCTTAGGC |
| KO-QF | CTGAAGAGTGGAAGCCTGAACG |
| KO-QR | CCCAAACGCCATTGTCTTCTGC |
| KS-QF | CCTCCTCATACCGATTTCCACAGC |
| KS-QR | TGTTTCACCACCATTGAGGACCTG |
| GA3ox1-QF | CCTTGTATAGGGCAGTGACATGG |
| GA3ox1-QR | CCGTACGGATGAAAGTGCCTTG |
| GA3ox2-QF | ATCTGTTCGGCTTTGTGCTCCTC |
| GA3ox2-QR | TGGACGCCACTTTGATCCTTGG |
| GA20ox1-QF | CGATGAATGGCGTTCCATCAGTCC |
| GA20ox1-QR | ACTACCGCTCTGTGTAGGCAAC |
| GA20ox2-QF | CCTCCATCAAGACAACGTCTCAGG |
| GA20ox2-QR | TTGGACTGATAGAGCGCCACTG |
| GA20ox3-QF | AATGCCGTGAGCACCCTTTCTC |
| GA20ox3-QR | TGCTCACACCTAGGCTCAATCC |
| GA20ox4-QF | ATGAGCAAGCTTTCCCTTAGTGTG |
| GA20ox4-QR | TCTTCCCACTCCAATACTCTCACC |
| ZF3-QF | AGGGTGAAGTGGAAGCACAAGC |
| ZF3-QR | GCGCTTGGAAAGATGAGAAGCG |
| Actin-QF | TGGAGGATCCATCCTTGCATCAC |
| Actin-QR | CGCCCTTTGAAATCCACATTTGCC |
| GA20ox4-amp1-QF | CTGGAACACGTTTTTCTCCTT |
| GA20ox4-amp1-QR | CTATTGACGTTCATGTTAGTGG |
| GA20ox4-amp2-QF | TGAAGAATATTCAAGTTAGTGAGAACG |
| GA20ox4-amp2-QR | TGGAGAAATCGAGAAGTTGG |
| GA20ox4-amp3-QF | TGGCATATAGCTAGAGGACACTG |
| GA20ox4-amp3-QR | TTGTCTCCTTCTCGTAAATACTCTT |
| GA20ox4-amp4-QF | TGTGAGGTAACATGGGCTGT |
| GA20ox4-amp4-QR | GAGGTGAAGGATGGAGCAGT |
| GA20ox4-amp5-QF | TTCCCACTCTGTGTTTTGGA |
| GA20ox4-amp5-QR | CAGTTGATGTTCAAAGAATACGC |
| GA20ox4-CK1-QF | CAGATGATGAAAAGCCATGC |
| GA20ox4-CK1-QR | TCACCGGAGAGATAGCCATT |
| GA20ox4-CK2-QF | TCATTCTTTAAACATAATTGTTTGC |
| GA20ox4-CK2-QR | TGACACATCAAACAAGTCTCACA |
