# Supplementary material for: SlZF3 regulates tomato plant height by directly repressing SlGA20ox4 in the gibberellic acid biosynthesis pathway
Source: Hortic Res. 2023 Feb 21;10(4):uhad025. doi: 10.1093/hr/uhad025 (PMC10116951; doi:10.1093/hr/uhad025)
Supplement: Web_Material_uhad025 [file web_material_uhad025.zip › Luo MS-Table S4.docx]

**Supplementary Data**

**Table S4** List of cis-elements that SlZF3 potentially binds to.

| Element name | Sequence 5’-3’ | Annotation |
| --- | --- | --- |
| ANAERO2CONSENSUS | AGCAGC | Anaerobic; |
| ARR1AT | NGATT | ARR1; Response regulator; |
| CAATBOX1 | CCAAT | Heat shock element; CONSTANS; |
| BOXLCOREDCPAL | ACCWWCC | MYB; R2R3 type; PAL: Elicitor; UV-B; |
| DOFCOREZM | AAAG | Dof; C4PEPC; PEPC; C4; leaf; shoot; |
